# *Mycobacterium bovis* Bacillus Calmette–Guérin Alters Melanoma Microenvironment Favoring Antitumor T Cell Responses and Improving M2 Macrophage Function

**DOI:** 10.3389/fimmu.2017.00965

**Published:** 2017-08-11

**Authors:** Ricardo D. Lardone, Alfred A. Chan, Agnes F. Lee, Leland J. Foshag, Mark B. Faries, Peter A. Sieling, Delphine J. Lee

**Affiliations:** ^1^Dirks/Dougherty Laboratory for Cancer Research, Department of Translational Immunology, John Wayne Cancer Institute, Providence Saint John’s Health Center, Santa Monica, CA, United States; ^2^Los Angeles Biomedical Research Institute at Harbor-UCLA Medical Center, Torrance, CA, United States; ^3^Division of Surgical Oncology, John Wayne Cancer Institute, Providence Saint John’s Health Center, Santa Monica, CA, United States; ^4^Melanoma Research Program, John Wayne Cancer Institute, Providence Saint John’s Health Center, Santa Monica, CA, United States

**Keywords:** cutaneous metastatic melanoma, intralesional bacillus Calmette–Guérin, melanoma microenvironment, antitumor immunity mechanisms, T cell response

## Abstract

Intralesional *Mycobacterium bovis* bacillus Calmette–Guérin (BCG) has long been a relatively inexpensive therapy for inoperable cutaneous metastatic melanoma (CMM), although intralesional BCG skin mechanisms remain understudied. We analyzed intralesional BCG-treated CMM lesions combined with *in vitro* studies to further investigate BCG-altered pathways. Since macrophages play a pivotal role against both cancer and mycobacterial infections, we hypothesized BCG regulates macrophages to promote antitumor immunity. Tumor-associated macrophages (M2) infiltrate melanomas and impair antitumor immunity. BCG-treated, *in vitro*-polarized M2 (M2-BCG) showed transcriptional changes involving inflammation, immune cell recruitment, cross talk, and activation pathways. Mechanistic network analysis indicated M2-BCG potential to improve interferon gamma (IFN-γ) responses. Accordingly, frequency of IFN-γ-producing CD4+ T cells responding to M2-BCG vs. mock-treated M2 increased (*p* < 0.05). Moreover, conditioned media from M2-BCG vs. M2 elevated the frequency of granzyme B-producing CD8+ tumor-infiltrating lymphocytes (TILs) facing autologous melanoma cell lines (*p* < 0.01). Furthermore, transcriptome analysis of intralesional BCG-injected CMM relative to uninjected lesions showed immune function prevalence, with the most enriched pathways representing T cell activation mechanisms. *In vitro*-infected MM-derived cell lines stimulated higher frequency of IFN-γ-producing TIL from the same melanoma (*p* < 0.05). Our data suggest BCG favors antitumor responses in CMM through direct/indirect effects on tumor microenvironment cell types including macrophages, T cells, and tumor itself.

## Introduction

Cutaneous melanoma is predicted to cause approximately 9,700 deaths from metastatic disease in the United States in 2017 (1). In-transit melanoma, metastasis is a pattern of lymphatic disease spread occurring in approximately 7% of patients (2). Most patients with in-transit melanoma can suffer significant locoregional toxicity due to painful, bleeding, or necrotic lesions, which may become superinfected. Moreover, they can develop stage IV systemic recurrence within 12–18 months (3). The diagnosis of distant metastatic disease carries a poor prognosis, with median survival rates less than 8 months prior to the era of checkpoint blockade inhibitors (4). *Mycobacterium bovis* bacillus Calmette–Guerin (BCG) is the best known agent for intralesional therapy of cutaneous melanoma metastases (5) and is a recommended therapeutic option in version 1.2017 NCCN Guidelines for inoperable stage III in-transit melanoma (6). Up to 90% of injected tumors regress (7–10) and surprisingly, up to 40% of patients receiving intralesional BCG experience regression of their non-injected tumors (Figure S1 in Supplementary Material) (11). The use of BCG in cancer is not limited to melanoma; in fact, instillation of BCG is used as an intravesical therapy for superficial bladder cancer (12) and as an adjuvant in some cancer vaccines (13).

When given intralesionally, BCG certainly encounters the tumor microenvironment, where tumor cells and their fate are dependent upon interactions with a variety of cells such as fibroblasts, endothelial cells, and immune cells (14). As core effectors of adaptive immunity, T cells play a key role in tumor immune surveillance within the tumor microenvironment, influencing melanoma patients’ survival (15, 16). For example, interferon gamma (IFN-γ)-expressing Th1 CD4+ T cells contribute to antitumor immunity by blocking neoangiogenesis (17) and promoting recruitment of tumor-killing cells including CD8+ T and NK cells (18). In addition to T cells and NK cells, macrophages (MΦs) are another important component of the tumor microenvironment (19), able to differentiate into a continuum of phenotypically and functionally different subpopulations in response to microorganisms and host mediators (20). For example, classically activated (M1) MΦs protect through tumoricidal activity, secretion of proinflammatory mediators and production of reactive nitrogen and oxygen species (21). In contrast, alternatively activated (M2) MΦs facilitate tumor progression by releasing anti-inflammatory mediators (22) and promoting angiogenesis (23). Indeed, the presence of M2-MΦs (resemblants of myeloid-derived suppressor cells (24) and tumor-associated MΦs-TAMs (19) found *in vivo*) or their markers is associated with decreased survival in melanoma patients (25, 26). Among these M2-MΦs markers is CD163, a member of the scavenger receptor cysteine-rich family class B that binds and internalizes hemoglobin–haptoglobin complexes (27). CD163 also works as erythroblast adhesion receptor, receptor for tumor necrosis factor-like weak inducer of apoptosis (TWEAK), and receptor for different pathogens, triggering signaling cascades that lead to secretion of immunomodulatory molecules (28).

Tumor microenvironment can influence MΦ phenotypes by providing M2-MΦ-polarizing conditions (29). In fact, some antitumor therapies aim to overcome these inhibitory conditions (30–33). Although the precise tumor elimination mechanisms triggered by intralesional BCG are unclear, there is rationale for immune-mediated events involving T cell participation (34, 35).

MΦs are the primary target for mycobacterial infections (36), may contribute to tumor regression and progression (37, 38), and have malleable phenotypes (39). On these basis, we hypothesized that intralesional BCG therapy might also alter M2-MΦ phenotypes to become better effectors of antitumor immunity. To explore intralesional BCG-promoted mechanisms of antitumor immunity, we investigated the phenotypic and functional changes induced by BCG on *in vitro*-polarized MΦs, in combination with transcriptome analysis of injected and uninjected melanoma lesions from patients undergoing intralesional BCG therapy. Our findings indicate that intralesional BCG may stimulate the melanoma tumor microenvironment to promote antitumor immune responses by altering macrophage and T cell activities at the site of disease.

## Materials and Methods

### Healthy Blood Donors and intralesional BCG Melanoma Patients

For this study, peripheral blood was acquired from healthy human donors enrolled in Alpha IRB- and Western IRB-approved protocols (Study ID LEED-HEALTHYVOLUNTEERS). For intralesional BCG melanoma patients, punch biopsies were obtained from in-transit metastases of melanoma patients enrolled in Alpha IRB-approved study (Study ID BCG_J 001) and receiving intralesional BCG (Table S4 in Supplementary Material). In all cases, written informed consent was obtained for all procedures. All subjects gave written informed consent in accordance with the Declaration of Helsinki.

### Reagents and Antibodies

*Mycobacterium bovis* TICE strain (Organon Teknika Corporation, Durham, NC, USA) is used by John Wayne Cancer Institute (JWCI) surgical oncologists for treating patients with intralesional BCG and was also used in all *in vitro* experiments. Generated in 1934 as a substrain from Pasteur Institute’s BCG, TICE is considered a “late strain” belonging to the tandem duplication-2 group IV strains, which also exhibit a deletion in the Region of Difference-2 (40). Strains in this group exhibit the highest levels of virulence in SCID mice and are also the more effective in protection against *Mycobacterium tuberculosis* challenge in BALB/c mice (41).

The following monoclonal antibodies were used: 215927 (anti-CD163, R&D Systems, Minneapolis, MN, USA), TuK4 (anti-CD14, Invitrogen, Waltham, MA, USA), 1-D1K and 7-B6-1 (anti-IFN-γ, Mabtech, Nacka Strand, Sweden), GB10 and GB11 (anti-GrB, Mabtech), 508A4A2, 508A7G8, and 508A3H12 (anti-IL1-β, Invitrogen), and appropriate isotype controls (R&D Systems and Invitrogen).

### MΦ Differentiation and Treatment

Peripheral blood mononuclear cells (PBMCs) were isolated from whole blood of healthy adult volunteers using Ficoll-Hypaque gradient centrifugation. CD14+ monocytes were enriched from PBMCs using negative selection (EasySep Human Monocyte Enrichment Kit, Stem Cell Technologies, Vancouver, BC, Canada). Monocytes (7.5 × 10^5^ cells/ml) were cultured in media [10% fetal calf serum (FCS) in RPMI 1640] supplemented with either rh-granulocyte macrophage colony-stimulating factor (GM-CSF) (50 U/ml) or rh-M-CSF (50 ng/ml) to induce differentiation into M1- or M2-MΦ, respectively, as previously described (42). On day 6 of culture, cells were infected with BCG at 0.18 MOI. Cells were harvested and washed 24 h after infection using warmed PBS-1 mM EDTA.

### LPS-Stimulated Cytokine Production

Concentration of harvested macrophages was adjusted to 10^5^ cells/ml. Triplicates of 200 μl of cells/well were plated for each condition in 96-well microtiter plates, in the presence of 10 ng/ml LPS or media alone. Cytokine production (IL10, IL12p40; human antibody pairs from Invitrogen) was measured from 24 h supernatants by ELISA following manufacturer’s instructions.

### T Cell Culture/Enrichment and Enzyme-Linked ImmunoSpot (ELISPOT) Assay

In parallel with MΦ differentiation and treatment, another aliquot of PBMCs was cultured (2 × 10^6^/ml) in media (10% pooled, heat inactivated human serum in RPMI 1640) supplemented with tetanus toxoid (5 µg/ml) and costimulatory reagent (CD28-CD49d, 20 µl/ml). On day 4, lymphoblasts were enriched using Ficoll-Hypaque gradient centrifugation and cultured for 3 more days with 8% FCS–2% human serum in RPMI 1640 supplemented with rhIL-2 (1 nM). On day 7, CD4+ T cells were enriched from the culture using negative selection (EasySep Human CD4+ T cell Enrichment Kit, Stem Cell Technologies, Vancouver, BC, Canada). These T cells were assayed in ELISPOT experiments with autologous MΦ.

For IFN-γ ELISPOT assays, Multiscreen-IP filter plates (96 wells; Millipore) were coated with anti-IFN-γ (1-D1K, 5 µg/ml) according to the manufacturer’s instructions. M1, M2, or M2-BCG were harvested and cultured (10^4^) in the plate overnight with autologous CD4+ T cells (5 × 10^4^), in the presence or the absence of Tetanus toxoid (10 µg/ml).

For BCG-infected melanomas, 10^4^ melanoma cell lines and 10^4^ tumor-infiltrating lymphocytes (TILs generated at JWCI using state-of-the-art techniques for adoptive cell transfer therapy (43) and kindly provided by Hitoe Torisu-Itakura) were incubated overnight in the plate (in all cases final volume per well was 200 µl). TILs had been previously enriched for CD8+ T cells from the TILs bulk culture by negative selection (EasySep Human CD8+ T cell Enrichment Kit, Stem Cell Technologies, Vancouver, BC, Canada). Cells were removed from the plate, and biotinylated detecting Ab was added (7-B6-1, 1 µg/ml) for 4 h. After washing, streptavidin-peroxidase conjugate (Pierce) was added to the plate for 1 h. To visualize cytokine-producing cells, substrate (3-amino-9-ethylcarbazole, Vector Labs) was added according to manufacturer instructions, and plates were incubated in the dark for 15 min. After washing and drying, ELISPOT plates were digitally scanned on an ImmunoSpot Series 3A Image Analyzer (Cellular Technology).

For granzyme B (GrB) ELISPOT assays, anti-GrB (GB10, 5 µg/ml) and biotinylated anti-GrB (GB11, 1 µg/ml) were used as coating and detecting antibodies in Multiscreen-IP filter plates, respectively. Melanoma target cells (5 × 10^3^) and effector TILs (10^4^) were incubated in the plates for 4 h. Remaining procedures and reagents were the same as for IFN-γ ELISPOT.

### Flow Cytometry

Aliquots of 10^5^ cells were blocked with a 1:1 mixture of human serum and 2% FCS in PBS, then stained using fluorescent labeled antibodies against different cell surface markers. A total of 10,000 gated events were acquired on a BD Biosciences FACSVerse flow cytometer and analyzed with FCS Express 4 software (*De Novo* Software, Los Angeles, CA, USA).

### RNA Isolation

For *in vitro* cell cultures, after removing cell culture supernatants, uninfected and BCG-infected macrophages were harvested in trizol. Chloroform (0.2 parts) was added; total RNA in upper phase of partition was precipitated with Isopropanol and washed with 80% ethanol. Further isolation steps for total RNA were continued using Qiagen RNeasy mini kit, according to manufacturer instructions. All isolated samples yielded RNA Integrity Number values ≥8 using an Agilent Technologies 2100 Bioanalyzer.

For melanoma tissue, skin biopsies from previously reported intralesional BCG-treated patients were processed as formerly described (44).

### cDNA Library Preparation and RNASeq

cDNA libraries were prepared using TruSeq RNA Library Preparation Kit v2 (RS-122-2001) according to the “TruSeq RNA Sample Preparation v2 Guide.” Briefly, purification of poly-A containing mRNA molecules from 300 ng of total RNA was performed using poly-T oligo attached magnetic beads. After fragmentation into small pieces using divalent cations under elevated temperature, the cleaved RNA fragments were copied into first strand cDNA using reverse transcriptase and random primers. Second strand cDNA synthesis was achieved using DNA Polymerase I and RNase H. These cDNA fragments went through further end repair process, single “A” base addition, and adapters ligation. The products were then purified and enriched with PCR to create the final cDNA library. Libraries were sequenced on Illumina HiSeq2000 at 50 million reads per sample and 1 × 50 read length. These procedures were performed by the UCLA Technology Center for Genomics and Bioinformatics (TCGB).^1^

### RNASeq Data Processing and Analysis

The reads obtained by RNASeq were processed and analyzed with specific tools piped together on Ubuntu operating system. Quality assurance of reads (GC content, adaptors and PHRED score assessment) was done with FastQC. Trimming to remove poor quality reads and adapters was performed using Trimmomatic. Read-mapping to the human reference genome hg19 and abundance estimation of genes and isoforms were done using Bowtie2 aligner within RSEM with default values. Estimated RPKM values were used to visualize clustering dendrograms, heatmaps, and the ordination plot. Raw counts were obtained from resulting BAM files using HTseq. Finally, differential gene expression was determined using the Wald test in DESeq2 package with raw counts as input. For the macrophage data, sample pairing was taken into account in the DESeq2 model. RNA sequencing data have been deposited for public access into NCBI’s Gene Expression Omnibus database-GEO (45) and Sequence Read Archive-SRA (46) and are accessible through accession numbers GSE90748 (GEO) and SRP094423 (SRA).

### Ingenuity Pathway Analysis (IPA)

The biological functions, canonical pathways, and networks most significantly represented in the different sets of genes were found using the “Core Analysis” function of IPA.^2^ Fold-change and *p*-value cutoffs were used in association with each uploaded set. The activation status of the functions/pathways was predicted using IPA “Upstream Regulator Analysis” tool by calculating a regulation *Z*-score and an overlap *p*-value, based on the number of known target genes of interest pathway/function, expression changes of these target genes and their agreement with literature findings. A significant activation (or inhibition) was considered with an overlap *p*-value ≤0.05 and IPA activation *Z*-score ≥2.0 (or ≤−2.0).

### Gene Ontology (GO) Enrichment Analysis

Enrichment analysis was performed for the lists of differentially expressed genes using the “Gene Ontology Consortium” enrichment analysis tool (47). Output list was summarized and visualized using the semantic similarity-based treemap tool of “REduce and VIsualize Gene Ontology” web server (REVIGO) (48). A *p*-value accounted for the probability that the linking of the genes list to each GO term was explained by chance alone. GO terms within the same process were grouped and equally colored, and the area values were set proportionally to −log10 (multiple hypothesis corrected) *p*-value for each term.

### Hierarchical Clustering and Principal Component Analysis (PCA)

Macrophage or intralesional BCG samples were classified into molecular subgroups by unsupervised hierarchical clustering in MultiExperimentViewer 4.9 (MeV), using Pearson correlation and average linkage. RPKM values were log2 transformed, mean centered per gene and clustered by average linkage, using leaf order optimization. PCA was used to visualize the differences between M1, M2, and M2-BCG samples.

### Statistical Analyses

Data are presented as means ± SEM unless indicated. We used Wilcoxon signed rank test and Mann–Whitney test with Prism 6 (GraphPad software, La Jolla, CA, USA). *p*-Values of less than 0.05 were considered significant. Degrees of statistical significance are presented as *p < 0.05; **p < 0.01; or ***p < 0.001.

## Results

### BCG Infection of *In Vitro*-Polarized M2-MΦs Induces Transcriptional Reprogramming Leading to an MΦ with Altered Phenotype and Functional Cytokine Response

**Figure 1:**
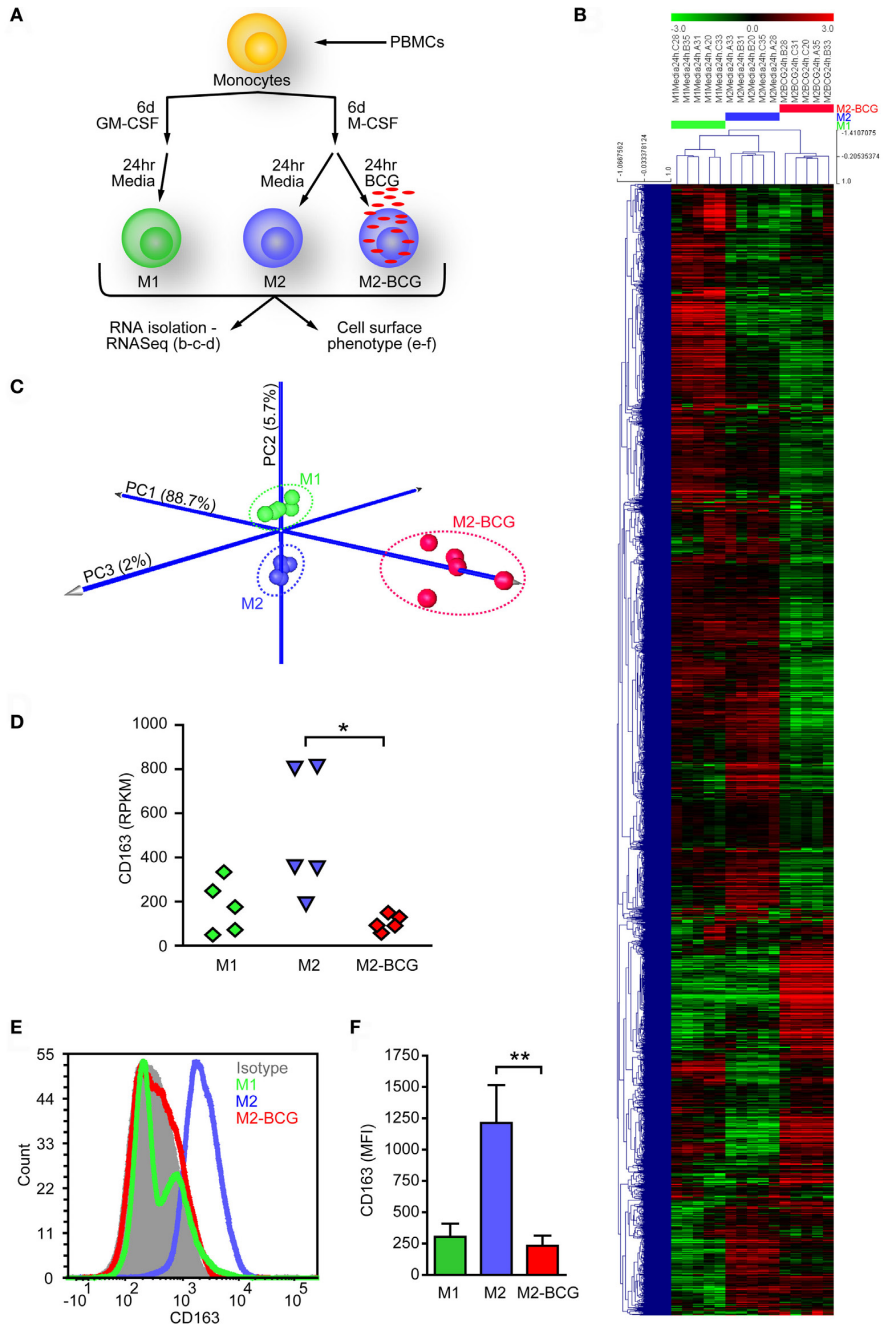
Bacillus Calmette–Guérin (BCG) infection of *in vitro*-polarized M2 macrophages elicits a different population of cells. **(A)** *In vitro* polarizing scheme used to study BCG effect on M2-MФs. Macrophages were polarized, infected, and harvested. From each condition, one fraction was used for RNA isolation and sequencing, while the other was assessed for CD163 cell surface expression. **(B)** Unsupervised hierarchical clustering of genes expressed in uninfected M1-MФs (M1), uninfected M2-MФs (M2), and BCG-infected M2-MФs (M2-BCG) at 24 h *post* infection (data filtered to the 5% of genes with highest variance, mean centering, Pearson correlation, and average linkage were used). **(C)** Principal component analysis plot of RNASeq data for M1, M2, and M2-BCG samples. 96.4% of variance in the combined dataset is captured in the analysis (88.7% in PC1—*X* axis, 5.7% in PC2—*Y* axis, and 2% on PC3—*Z* axis). **(D)** CD163 transcript levels (in RPKM) from M2 were reduced upon BCG infection. **(E)** Representative plot showing BCG-induced downregulation of CD163 surface levels on M2. **(F)** Summary of data from *n* = 6 healthy donors.

Previous reports have shown that BCG can alter the function and surface marker profiles of dendritic cells (49, 50). To determine whether BCG can alter the phenotype of M2-MΦs, we performed transcriptome analyses of polarized MΦs. M2-MΦs were infected with BCG (M2-BCG) or left untreated (M2), and cells were collected 24 h after infection. Uninfected M1-MΦs (M1) were also evaluated for comparison (Figure 1A). BCG infection induced broad changes in mRNA profiles, where expression of 611 genes was significantly modified at least threefold (*p* < 0.01, Table S1 in Supplementary Material). Unsupervised hierarchical clustering (Figure 1B) and PCA (Figure 1C) clearly separated the three different cell populations. Fivefold reduction of CD163 transcript levels from M2 upon BCG infection was evident (Figure 1D) and further verified by flow cytometry analysis of CD163 surface levels (Figures 1E,F). M2-BCG also produced 94% less IL10 and 500% more IL12p40 in response to LPS compared to M2 (Figure S2 in Supplementary Material).

### M2-BCG Gene Signature Shows Enrichment for Immune Cell Recruitment and Cytokine Signaling Pathways

**Figure 2:**
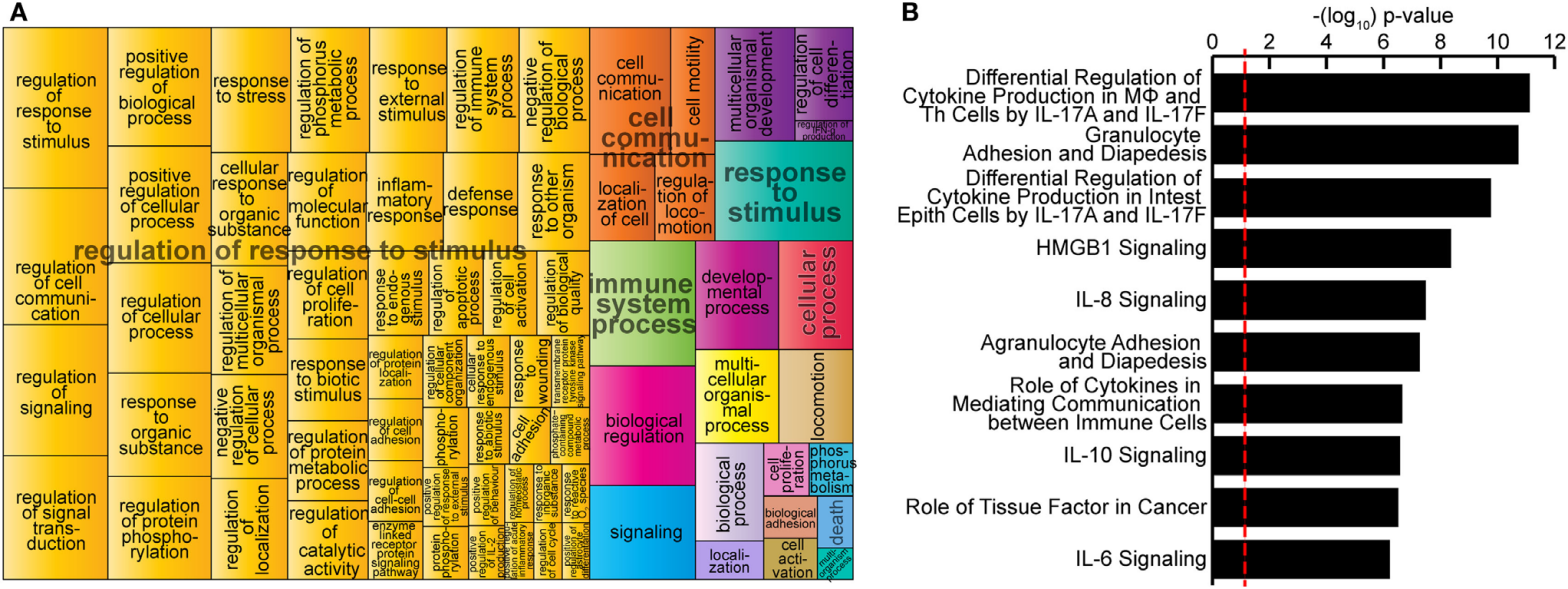
Bacillus Calmette–Guérin (BCG)-induced extensive transcriptional changes on *in vitro*-polarized macrophages. **(A)** Most highly enriched Gene Ontology (GO) terms according to Gene Ontology Consortium and REduce and VIsualize Gene Ontology web server algorithms (see Materials and Methods) for genes significantly upregulated in M2-BCG compared to M2 at 24 h after infection. GO terms are represented by tiles, grouped and colored according to semantic similarities to other GO terms. Tile areas are proportional to −log10 *p*-value for each term. **(B)** Top-10 canonical pathways identified by Ingenuity Pathway Analysis from the list of differentially expressed genes at 24 h after infection. Pathways are ranked by multiple hypothesis corrected *p*-values.

Bacillus Calmette–Guérin reprogramming of M2 was further demonstrated by pathway analysis of gene expression profiles using GO terms according to the Gene Ontology Consortium and REVIGO algorithms (see Materials and Methods). GO analysis mapped 312 significantly enriched GO biological functions (B-H *p* < 0.05, Table S2 in Supplementary Material) for genes significantly upregulated in M2-BCG compared to M2 at 24 h after infection. REVIGO analysis of these functions grouped many of them under the “Regulation of response to stimulus” term (Figure 2A), which describes changes in state or activity (movement, secretion, enzyme production, gene expression, etc.) as a result of a stimulus (47). IPA identified 146 significantly enriched canonical pathways (B-H *p* < 0.05, Table S3 in Supplementary Material). Most of the top-10 canonical pathways were related to regulation of cytokine production, to recruitment and activation of different immune cells and to signaling of various cytokines (Figure 2B). Therefore, BCG triggers a change in the functional pathways expressed by M2-MΦs.

### Mechanistic Network from Upstream Regulator Analysis Connects BCG-Induced Gene Expression Changes with IFN-γ

**Figure 3:**
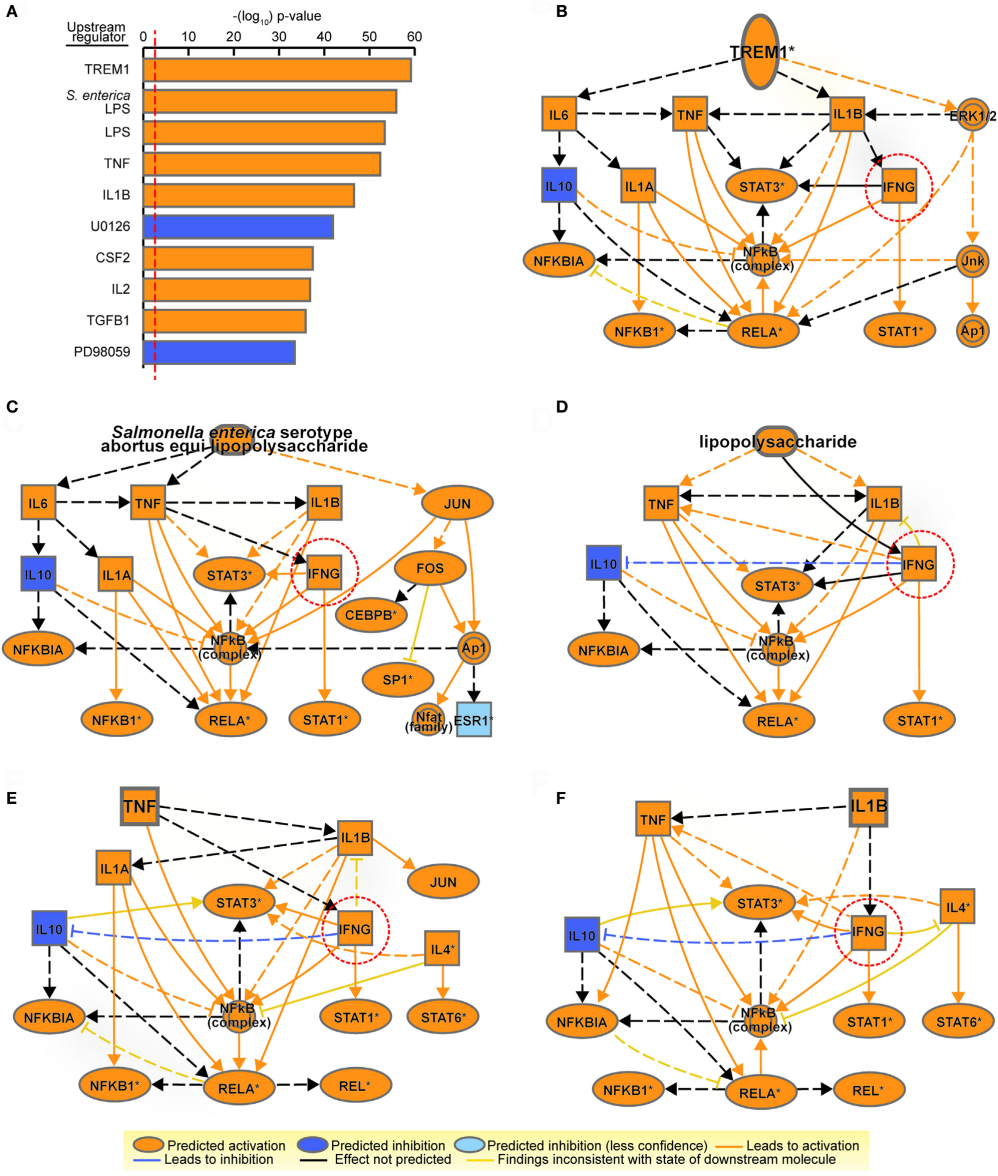
Mechanistic networks for the most significant upstream regulators identified in M2-bacillus Calmette–Guérin (BCG) indicate a potential for influencing T cells. **(A)** Top-10 upstream regulators predicted by Ingenuity Pathway Analysis from the list of differentially expressed genes in M2-BCG compared to M2. Regulators are ranked by multiple hypothesis corrected overlap *p*-values. Predicted activation status is shown in orange (for activated) or in blue (for inhibited). *S. enterica* LPS, *Salmonella enterica* serotype abortus equi lipopolysaccharide; U0126, succinonitrile bis(amino(*o*-aminophenylthio)methylene); PD98059, 2′-amino-3′-methoxyflavone. **(B–F)** Mechanistic networks for the top five most significant upstream regulators: **(B)** triggering receptor expressed on myeloid cells 1 (TREM1); **(C)** *S. enterica* serotype abortus equi lipopolysaccharide; **(D)** lipopolysaccharides; **(E)** TNF; **(F)** IL1B. A potential for influence on T cells is indicated by the predicted presence of IFNG (dotted circle) in all five networks. Nodes and edges are represented according to their predicted activation status.

Upstream regulator analysis by IPA identified the upstream transcriptional regulators that explain the gene signature upregulated upon BCG. The top upstream activated regulator predicted was “Triggering receptor expressed on myeloid cells 1” (TREM1), a macrophage/neutrophil receptor that amplifies inflammation induced by stimulation of pattern-recognition receptors. In addition to TREM1, inflammatory cytokine genes such as TNF and IL1β were also identified as important upstream regulators (Figure 3A). Consistently, IL1β protein levels were much more elevated (~4,700 pg/ml) in M2-BCG than in M2 (~300 pg/ml) culture supernatants (*p* < 0.01). Mechanistic networks for the top five upstream regulators (comprising a majority of innate immunity-related components) contained IFNG (Figures 3B–F). IFN-γ, a very important mediator in antitumor immunity (51), suggested a potential link between innate and adaptive immunity.

### BCG-Treated Macrophages Promote IFN-γ Production

**Figure 4:**
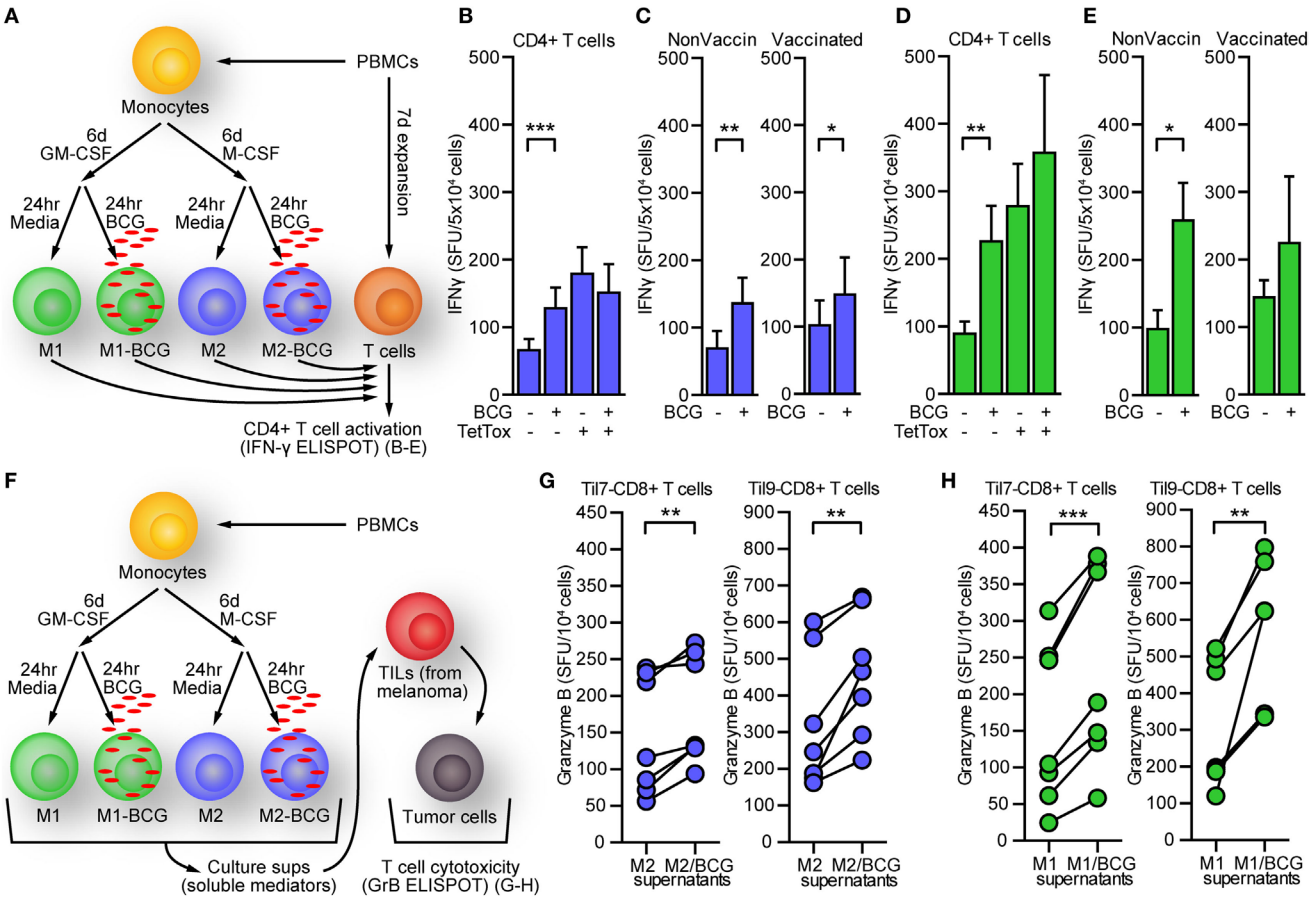
Bacillus Calmette–Guérin (BCG)-treated macrophages induce greater activation on T cells in the absence of antigen, and their supernatant can condition tumor-infiltrating lymphocytes (TILs) to improve antitumor immunity. **(A)** *In vitro* polarization, infection, and enzyme-linked immunoSpots (ELISPOTs). Macrophages were polarized, infected, and harvested as explained in the Section “Materials and Methods.” Meanwhile, another fraction of peripheral blood mononuclear cells (PBMCs) was cultured for 7 days in 10% fetal calf serum media-RPMI (in the presence of tetanus toxoid) and subsequently enriched for CD4+ T cells by negative selection. These T cells were assayed in ELISPOT experiments with macrophages. **(B)** M2-BCG (blue bars) increased significantly the frequency of interferon gamma (IFN-γ)-producing autologous T cells in an antigen non-specific manner. **(C)** The response was observed regardless of BCG vaccine status (*p < 0.05, **p < 0.01, Wilcoxon signed rank test). **(D)** M1-BCG (green bars) induced an even greater enhancement on the frequency of IFN-γ-producing autologous T cells, an effect not favored by previous BCG vaccination **(E)**. **(F)** Supernatants from polarized, BCG-infected macrophages were used to condition TILs before targeting autologous melanoma cells. **(G)** Supernatants from M2-BCG enhanced granzyme B (GrB) release from TILs in response to autologous tumor cells. **(H)** Likewise, M1-BCG supernatants also increased GrB release. Number of GrB SFU from independent experiments with supernatant sets from seven different donors are shown as the mean ± SEM. Results with two different pairs of TILs and autologous tumor cells are shown (**p < 0.01, ***p < 0.001, Wilcoxon signed rank test). SFU, spot forming units. Error bars indicate SEM.

To determine whether M2-BCG could increase IFN-γ production from T cells, we evaluated T cell responses using an IFN-γ-based ELISPOT assay in which autologous T-cells were cocultured with different MΦs populations (Figure 4A). T cells cultured with autologous M2-BCG had a 100% higher frequency of IFN-γ producing cells than those cultured with M2, in a non-antigen-specific manner (Figure 4B). Live and heat-killed (30′ at 75°C) BCG caused similar effect (data not shown). Differences were not attributable to BCG-vaccination status, since MΦs and T cells from BCG non-vaccinated and BCG-vaccinated individuals showed similar IFN-γ responses (Figure 4C). When T cells were cultured with autologous M1-BCG the frequency changes on IFN-γ producing cells were even greater (Figure 4D). Previous BCG vaccination did not seem to favor this effect (Figure 4E).

### Supernatants from M2-BCG Cell Cultures Promote TILs Release of GrB in Response to Tumor Cells

Since M2-BCG upregulated several cytokine genes, we hypothesized BCG infection of M2 may result in secretion of soluble factors that enhance T cell antitumor responses. M2 were infected with BCG and their supernatants collected at 24 h. After adding these supernatants to cocultures of autologous TILs and melanoma tumor cells previously isolated from metastatic melanoma, GrB release was measured by ELISPOT (Figure 4F). Using two different pairs of TILs and autologous melanoma cells (see Materials and Methods), we found that supernatants from seven different M2-BCG donors enhanced GrB release from TILs by ~25% in response to their autologous tumor cells (Figure 4G). These changes were also observed when testing M1-BCG supernatants (Figure 4H).

### Gene Expression Profiles from BCG-Injected Cutaneous Metastatic Melanoma (CMM) Show Comparatively Higher Expression of T Cell Activation Signatures

**Figure 5:**
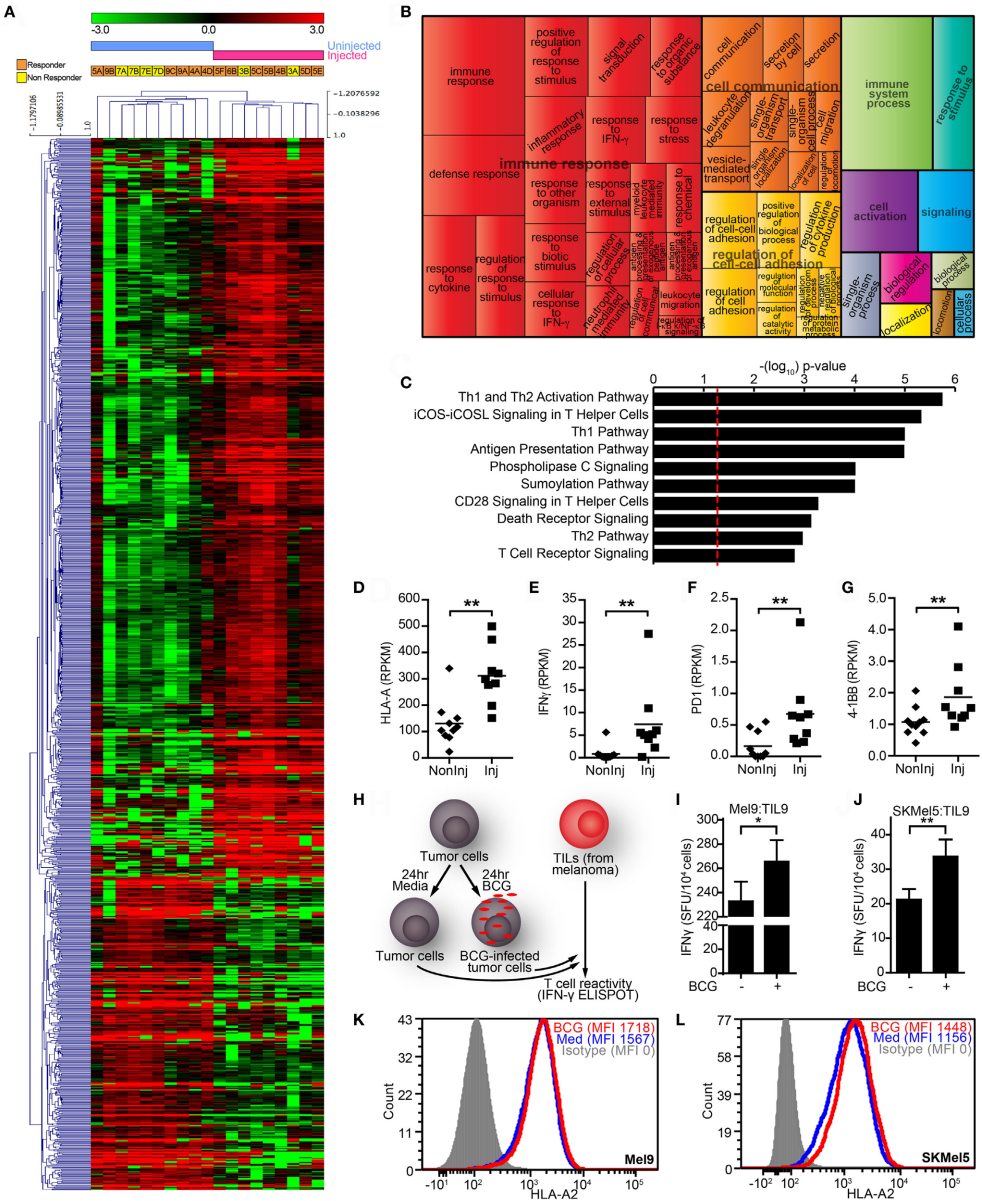
Intralesional BCG therapy on melanoma patients. **(A)** Unsupervised hierarchical clustering of RNASeq data for genes differentially expressed in bacillus Calmette–Guérin (BCG)-injected vs. uninjected melanoma lesions biopsied from different intralesional BCG patients (data filtered to the 1,121 most differentially expressed genes by fold change ≥2 or ≤−2 and *p*-value <0.01; mean centering, Pearson correlation, and average linkage were used). Responders (orange) and non-responders (yellow) to intralesional BCG treatment are indicated for each sample (see Table S4 in Supplementary Material for full detail). **(B)** Most highly enriched Gene Ontology (GO) terms according to Gene Ontology Consortium and REduce and VIsualize Gene Ontology web server algorithms (see Materials and Methods) for genes significantly upregulated in BCG-injected lesions. GO terms are represented by tiles, grouped and colored according to semantic similarities to other GO terms. Tile areas are proportional to −log10 *p*-value for each term. **(C)** Top-10 canonical pathways identified by Ingenuity Pathway Analysis from the list of differentially expressed genes. Pathways are ranked by multiple hypothesis corrected *p*-values. **(D)** HLA-A transcript levels (in RPKM) from BCG-injected lesions were significantly higher than uninjected ones. **(E)** A similar finding was observed for interferon gamma (IFN-γ) transcript levels (in RPKM). Consistently, increased levels (in RPKM) of T cell activation marker transcripts **(F)** PD1 and **(G)** 4-1BB were also verified. The horizontal line represents the population mean **(H)** simplified *in vitro* scheme used to study BCG-mediated changes in melanoma cell recognition by tumor-infiltrating lymphocytes (TILs). HLA-A2+ tumor cells infected with BCG (0.18 MOI) were harvested and washed after 24 h of infection (to remove unbound BCG), then used as target cells in IFN-γ-enzyme-linked immunoSpot (ELISPOT) plates paired with HLA-A2-restricted TILs (effector-to-target ratio of 2:1). **(I)** BCG infection of autologous HLA-A2+ melanoma cell line (Mel9) enhances IFN-γ production on HLA-A2-restricted TILs (Til9) from a melanoma patient. **(J)** Similar effect was observed when using a heterologous HLA-A2+ cell line (SKMel5) as target for the same HLA-A2-restricted TILs. IFN-γ spot forming units (SFU) are shown as the mean ± SEM from eight (autologous melanoma) or three (SKMel5) independent IFN-γ ELISPOT experiments (*p < 0.05, **p < 0.01, Wilcoxon signed rank test). **(K,L)** BCG increased levels of HLA-A2 on melanoma cells, which may account for the enhanced IFN-γ production.

To investigate the effects of intralesional BCG therapy on CMM *in vivo*, we analyzed the transcriptome of BCG-injected vs. uninjected CMM lesions. Unsupervised hierarchical clustering separated the tissues in accordance to whether they had been injected or not (Figure 5A, see legend for parameter details). BCG-injected tumors showed significantly higher expression of 175 genes at least twofold (*p* < 0.01, Table S5 in Supplementary Material). Pathway analysis using GO exploration mapped 403 significantly enriched GO biological functions in injected vs. uninjected tumors (B-H *p* < 0.05, Table S6 in Supplementary Material). REVIGO analysis of the top 200 GO functions grouped many of them under the “immune response” and “immune system process” terms (Figure 5B). IPA identified 72 significantly enriched canonical pathways (B-H *p* < 0.05, Table S7 in Supplementary Material). The top-10 canonical pathways were largely representative of T cell activation mechanisms, such as “Th1 and Th2 Activation Pathway,” “iCOS-iCOSL Signaling in T Helper Cells” and “Phospholipase C Signaling” (Figure 5C). Consistently, BCG-injected lesions presented significantly higher transcript levels (in RPKM) of genes involved in these mechanisms, such as HLA-A (Figure 5D), IFNG (Figure 5E), PD1/PDCD1 (Figure 5F), and 4-1BB/TNFRSF9 (Figure 5G). To determine whether T cells generate a stronger immune response against BCG-treated melanoma cell lines, we cultured HLA-A2+ melanoma cell lines with BCG *in vitro* and after 24 h, washed the tumor cells and cultured them with TILs isolated from an HLA-A2+ patient’s CMM (Figure 5H). We found that autologous (MEL9) and allogeneic (SKMel5) melanoma cell lines treated with BCG stimulated 14–62% more TILs clones to secrete IFN-γ (Figures 5I,J, respectively). The observed increase in HLA-A2 levels of BCG-treated melanoma cells (Figure 5K, Mel9; Figure 5L, SKMel5) can account (at least in part) for this behavior. Active mechanisms by live BCG did not seem necessary for IFN-γ enhancement, since Mel9 treated with heat-killed BCG exerted a similar effect, as did ligands for two well-established BCG-engaging receptors, TLR2 and TLR9 (Supplementary Figure S3 in Supplementary Material).

## Discussion

In the late 1800s, Coley pioneered intralesional immunotherapy of sarcomas using bacterial components (52). Decades later, Coley’s vision inspired Morton and colleagues to use intralesional BCG for metastatic melanoma treatment (9). Regimens at that time used high BCG doses and triggered adverse side effects that weakened enthusiasm for the therapy (53, 54). Efforts from limited number of centers continued to refine the approach that lead to current therapeutic benefits observed with much lower doses and with mild adverse events (55, 56). However, the mechanisms by which intralesional BCG contributes to tumor regression in CMM remain unclear, demanding additional investigation. Thus, we investigated the role of BCG on MΦs and T cells, two key immune cell types present in the tumor microenvironment. We found that BCG reprogrammed M2-MΦs (a cell type linked to poor survival in several tumors, including melanoma) to become a transcriptionally and functionally distinct cell population. Mechanistic network analysis unveiled a connection between these changes and IFN-γ. Interestingly, we found M2-BCG was able to enhance IFN-γ production from CD4+ T cells. Finally, transcriptional analysis of BCG-injected vs. uninjected melanoma lesions from intralesional BCG patients’ biopsies indicated enrichment in T cell activation pathways, a feature we could also verify on a simplified *in vitro* scheme for BCG treatment of tumor cells.

A growing body of epidemiologic, clinical, and immunologic evidence indicates that certain microorganisms can exert beneficial non-specific effects against other pathogen/disease/malignancies, by potentiation of mechanisms like innate immune memory (through epigenetic remodeling of innate immunity, or “trained immunity”) and cross-reacting lymphocytes (57). Indeed, innate immune pathways are activated sooner than adaptive immune cell-mediated responses upon exposure to BCG (58), leading to epigenetic reprogramming of circulating monocytes exhibiting an enhanced and lasting phenotype (59). Consistently, we found extensive transcriptional changes in M2-BCG compared to M2. Although M2-BCG gene expression signatures were distinct from M1 and M2, when compared to M2 they showed comparatively higher expression of M1 genes such as cytokines TNF, IL6, IL1B, chemokines CXCL8, the chemokine receptor CCR7, and regulators FOS/JUN, EGR1, and EGR3 (60). This is relevant in light of recent observations by Falleni and coworkers (26) where M2-recruited TAMs overwhelm M1 accumulation in all stages of MM progression, thus suggesting immune cancer therapies should focus on conversion of protumorigenic M2 macrophage characteristics into M1 (or somewhat similar) antitumoral phenotype. However, it is also possible that M1 macrophages present in the lesions may also become activated upon intralesional BCG and contribute to the antitumor response. Although for our study the scarce amount of punch biopsy tissue obtained after pathology evaluation was exhausted during RNA isolation for RNASeq experiments, further studies on new samples are warranted to address the effect of BCG on M1 and M2 macrophage markers in the context of intralesional BCG.

Among the top enriched pathways observed in M2-BCG was “HMGB1 signaling.” Dying cells can expose various intracellular molecules like HMGB1 (61), calreticulin (62), phosphatidylserine (63), and nucleic acids and their degradation products (64), in a cell death mechanism known as “immunogenic cell death” (ICD) (65). ICD stimulates an immune response against these dead-cell antigens acting as damage-associated molecular patterns to engage various pattern-recognition receptors (PRR) and activate myeloid and lymphoid immune cells (66). BCG can trigger mechanisms that mimic HMGB1 effect: by inducing caspase-independent cell death in tumor cells, together with release of HMGB1 (67), and also by displaying ligands that predominantly bind to human TLR2 and TLR4 (49). Meanwhile, mechanistic network analysis of M2-BCG gene signatures predicted that the top upstream regulator activated by BCG was TREM1. This receptor, which sustained expression can be induced by BCG cell wall components (68), is known to interact with (or to be part of) toll-like receptor (TLR) 4 complex, and to amplify its signaling (69). All these findings are consistent with the ability of M2-BCG to positively influence IFN-γ responses on T cells (suggested by mechanistic network analyses and confirmed *in vitro*), resembling M1 antitumor ability (39). Interestingly, we found this enhancement in IFN-γ response occurred regardless of BCG-vaccination status of the donors. This phenomenon, also reported when transcriptionally profiling skin biopsies of tuberculin skin test across individuals with or without prior BCG vaccination (70), indicates the observed response might not be dependent upon classical memory immune responses.

Another interesting finding was that the soluble fraction produced by M2-BCG enhanced the release of the cytolytic molecule GrB from T cells responding to autologous melanoma cells. IL1β has been described to increase proportion of GrB-expressing CD8 T cells (71, 72). Although we found IL1β levels increased in supernatants from M2-BCG, blocking of IL1β did not prevent M2-BCG conditioned medium from increasing GrB release in our TILs-melanoma short-term cocultures (not shown). Further work is warranted to determine the relevant soluble factors involved in promoting antitumor activities of TIL, as it has been suggested for others (e.g., IL6 on long term *in vitro* settings) (73).

Intratumoral presence of activated, cytokine-producing TILs and preserved HLA class I expression have been associated with favorable outcome and prolonged patient survival in melanoma (74–76). Although examining different time points, the modifications we observed in an *in vivo* setting such as injected vs. uninjected melanoma lesions of patients receiving intralesional BCG were consistent with the findings obtained *in vitro*. BCG injection promoted transcriptional differences largely represented by enrichment in T cell activation mechanisms. BCG-injected lesions presented increased transcripts levels of (among others) HLA-A, IFNG, PD1, and 4-1BB, a finding compatible with the enhanced production of IFN-γ observed on HLA-A2-restricted TILs challenged with HLA-A2+ tumor cells preexposed to BCG *in vitro*. Research in bladder cancer shows BCG attachment to tumor cells, internalization, processing of the mycobacterium, and presentation of BCG antigens to T cells would play a crucial role in activation of BCG-mediated antitumor activity (77, 78). Whether or not melanoma cells also internalize BCG remains an open question that warrants further investigation. Nevertheless, BCG treatment made melanoma cells able to better activate T cells, showing increased expression of HLA-A2 and reactivity to ligands of TLRs typically engaged by BCG. In a context of immune surveillance activated after BCG therapy, immunoselection and elimination of tumor cells with increased HLA class I is a likely event. However, this might also allow further outgrowth of cancer cells with low levels of HLA, as it has been suggested for BCG therapy in bladder cancer (79). Although additional studies are required, this provides a potential explanation for why some BCG-injected melanoma lesions do not regress.

Health-care costs of managing melanoma are set to expand quickly, fueled by aging demographics, health price inflation, more expensive health services and new technologies (80). For example, a new oncolytic viral therapy (talimogene laherparepvec, or T-VEC) was recently approved in the USA for local treatment of unresectable cutaneous, subcutaneous and nodal melanoma lesions. T-VEC is a genetically modified, live, attenuated, herpes simplex virus type 1 that selectively replicates within tumors and produce GM-CSF to enhance systemic antitumor immune responses (81). However, this costly therapy has not yet been shown to improve overall survival or to have an effect on visceral metastases (82), unlike intralesional BCG (83). The BCG effects observed in different settings shed light on mechanisms consistent with intralesional BCG-induced modifications of melanoma microenvironment and promotion of antitumor T cell responses. Additional contributing mechanisms might involve other cell types such as dendritic cells, neutrophils and γδ T cells (44, 84, 85). A better understanding of the mechanisms and final effector pathways involved in intralesional BCG therapy may suggest novel strategies for improved, less expensive therapies. Some of them might include recombinant BCG secreting functional cytokines, as it has been tested for murine models of bladder cancer (86–88) and melanoma (89).

## Ethics Statement

For this study, peripheral blood was acquired from healthy human donors enrolled in Alpha IRB- and Western IRB-approved protocols (Study ID LEED-HEALTHYVOLUNTEERS). For intralesional BCG melanoma patients, punch biopsies were obtained from in-transit metastases of melanoma patients enrolled in Alpha IRB-approved study (Study ID BCG_J 001) and receiving intralesional BCG (Table S4 in Supplementary Material). In all cases, written informed consent was obtained for all procedures. All subjects gave written informed consent in accordance with the Declaration of Helsinki.

## Author Contributions

Contributions of the authors were as follows: RL, AL, PS, and DL designed the experiments. RL and AL performed the experiments. RL and AC performed bioinformatics and statistical analysis. RL, PS, and DL recruited healthy donors and wrote the manuscript. LF, MF, and DL recruited patients. LF and MF treated patients and collected patients’ clinical information. AC, AL, LF, and MF critically reviewed the manuscript. All the authors approved the final version of the manuscript.

## Conflict of Interest Statement

The authors state that research was conducted in the absence of any commercial or financial relationships that could be construed as a potential conflict of interest. The reviewer, EL, and handling editor declared their shared affiliation, and the handling editor states that the process nevertheless met the standards of a fair and objective review.

## Footnotes

^1^http://pathology.ucla.edu/tcgb.

^2^http://ingenuity.com.

## Supplementary Material

Figure S1 Intralesional BCG therapy on melanoma patients. Tumors treated with intralesional BCG go through macroscopic changes like inflammation, flattening, and eventual regression over time.

Figure S2 Bacillus Calmette–Guérin (BCG)-induced functional changes on *in vitro*-polarized macrophages. **(A)** *In vitro* polarizing scheme used to study BCG effect on M2-MΦs. **(B)** BCG infection decreased IL10 production on M2-MΦ upon LPS stimulation. **(C)** BCG infection increased IL12p40 secretion stimulated by LPS.

Figure S3 Melanoma cell line treatment with bacillus Calmette–Guérin (BCG) or toll-like receptor (TLR) ligands enhance interferon gamma (IFN-γ) production on autologous TILs. Aliquots of melanoma cell line (Mel9) were treated overnight with the following conditions: culture media only; BCG (MOI 0.18); heat-killed BCG (bacteria for 30′ at 75°C, amount equivalent to MOI 0.18); 19 kDa (TLR2 ligand, 10 µg/ml), LPS (TLR4 ligand, 0.1 µg/ml), CpG (TLR9 ligand, 1 µg/ml). The following day, Mel9 cells were harvested, washed, and tested in IFN-γ enzyme-linked immunoSpot assay with autologous CD8+ Til9 as described in the Section “Materials and Methods.” BCG, dead BCG, TLR2_lig_, and TLR9_lig_ treatments enhanced IFN-γ production on autologous TILs compared to untreated Mel9 (**p* < 0.05, paired *t* test).

## Abbreviations

BCG, bacillus Calmette–Guérin; CMM, cutaneous metastatic melanoma; ELISPOT, enzyme-linked immunoSpot; FCS, fetal calf serum; GO, Gene Ontology; GrB, granzyme B; IFN-γ, interferon gamma; IL, interleukin; IPA, Ingenuity Pathway Analysis; M1, classically activated Mϕs; M2, alternatively activated Mϕs; Mϕs, macrophages; PCA, principal component analysis; REVIGO, REduce and VIsualize Gene Ontology web server; RPKM, reads per kilobase per million mapped reads; TILs, tumor-infiltrating lymphocytes; TLR, toll-like receptor.
