# Supplementary figures and images for: Mycobacterium bovis Bacillus Calmette–Guérin Alters Melanoma Microenvironment Favoring Antitumor T Cell Responses and Improving M2 Macrophage Function
Source: Front Immunol. 2017 Aug 11;8:965. doi: 10.3389/fimmu.2017.00965 (PMC5554507; doi:10.3389/fimmu.2017.00965)

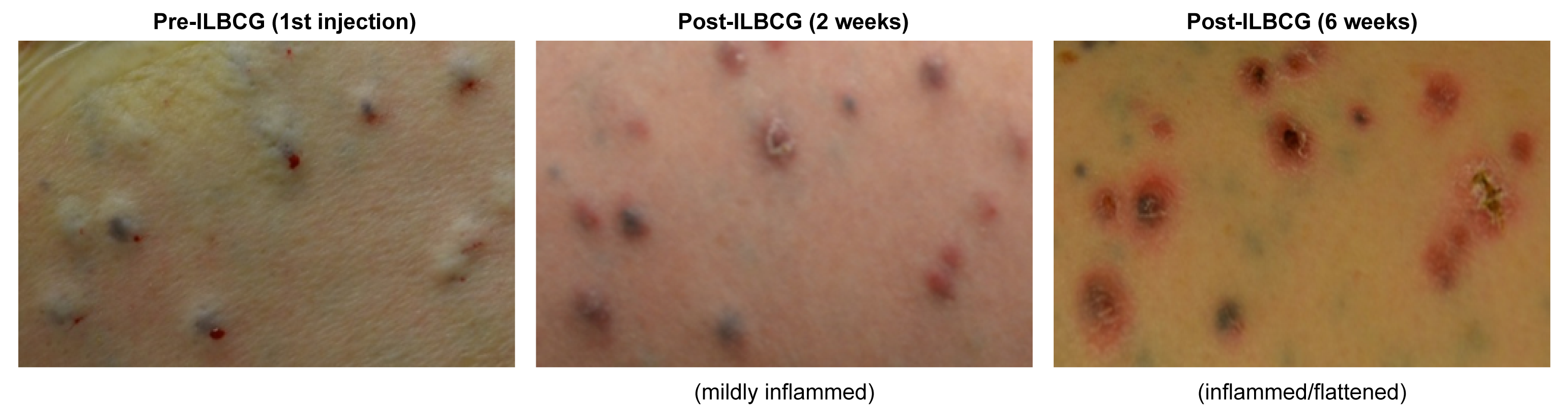

Supplement: Figure S1 — Intralesional BCG therapy on melanoma patients. Tumors treated with intralesional BCG go through macroscopic changes like inflammation, flattening, and eventual regression over time. [file image_1.tif]

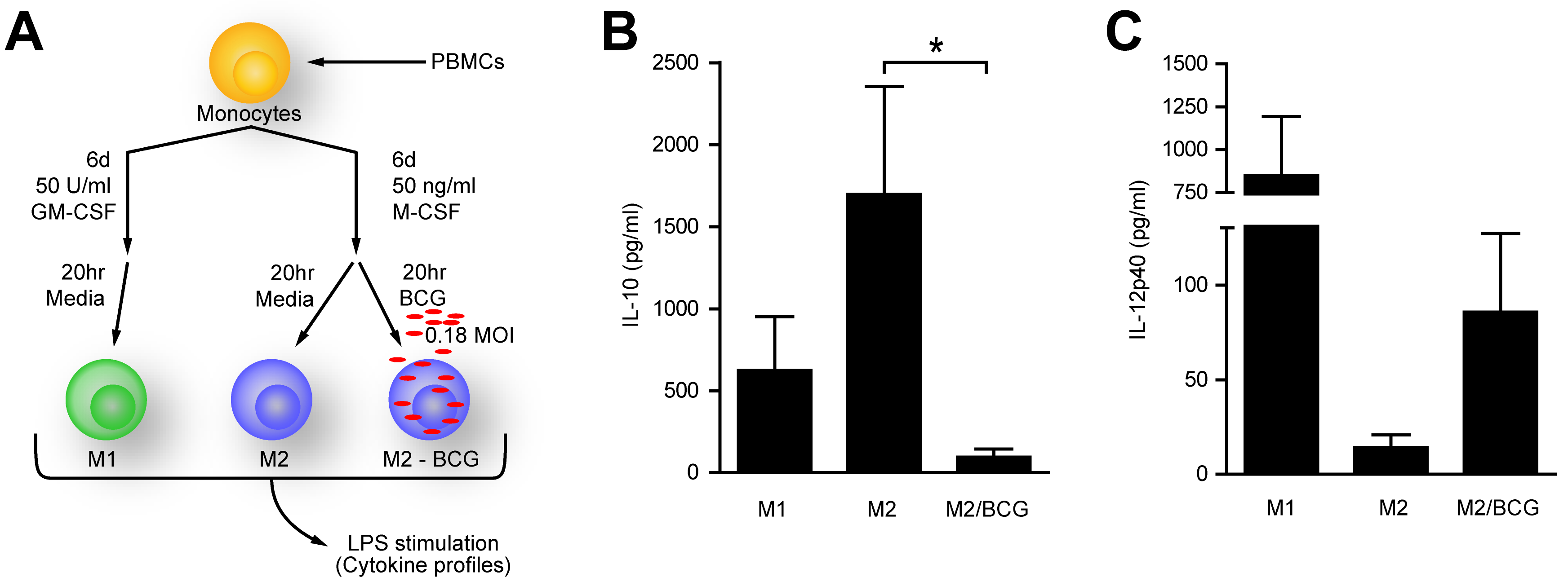

Supplement: Figure S2 — Bacillus Calmette–Guérin (BCG)-induced functional changes on in vitro-polarized macrophages. (A) In vitro polarizing scheme used to study BCG effect on M2-MΦs. (B) BCG infection decreased IL10 production on M2-MΦ upon LPS stimulation. (C) BCG infection increased IL12p40 secretion stimulated by LPS. [file image_2.tif]

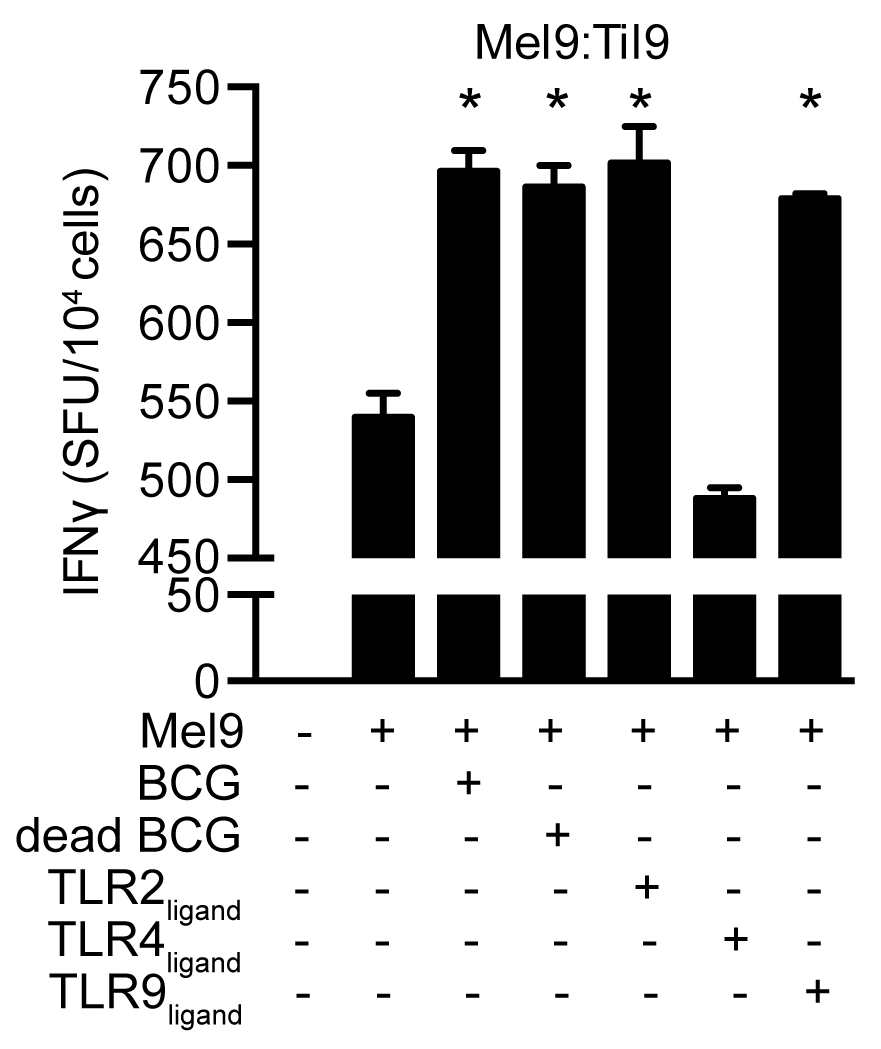

Supplement: Figure S3 — Melanoma cell line treatment with bacillus Calmette–Guérin (BCG) or toll-like receptor (TLR) ligands enhance interferon gamma (IFN-γ) production on autologous TILs. Aliquots of melanoma cell line (Mel9) were treated overnight with the following conditions: culture media only; BCG (MOI 0.18); heat-killed BCG (bacteria for 30′ at 75°C, amount equivalent to MOI 0.18); 19 kDa (TLR2 ligand, 10 µg/ml), LPS (TLR4 ligand, 0.1 µg/ml), CpG (TLR9 ligand, 1 µg/ml). The following day, Mel9 cells were harvested, washed, and tested in IFN-γ enzyme-linked immunoSpot assay with autologous CD8+ Til9 as described in the Section “Materials and Methods.” BCG, dead BCG, TLR2lig, and TLR9lig treatments enhanced IFN-γ production on autologous TILs compared to untreated Mel9 (*p < 0.05, paired t test). [file image_3.tif]
